# RIPK3 cleavage is dispensable for necroptosis inhibition but restricts NLRP3 inflammasome activation

**DOI:** 10.1038/s41418-024-01281-x

**Published:** 2024-03-21

**Authors:** Hong Tri Tran, Tobias Kratina, Auriane Coutansais, Dominika Michalek, Benjamin M. Hogan, Kate E. Lawlor, James E. Vince, John Silke, Najoua Lalaoui

**Affiliations:** 1 Peter MacCallum Cancer Centre, Melbourne, VIC Australia; 2 Sir Peter MacCallum Department of Oncology, University of Melbourne, Melbourne, VIC Australia; 3 Department of Anatomy and Physiology, University of Melbourne, Parkville, VIC Australia; 4 Centre for Innate Immunity and Infectious Diseases, Hudson Institute of Medical Research, Clayton, VIC Australia; 5 Department of Molecular and Translational Science, Monash University, Clayton, VIC Australia; 6 The Walter and Eliza Hall Institute of Medical Research, Parkville, VIC Australia; 7 Department of Medical Biology, University of Melbourne, Parkville, VIC Australia

**Keywords:** Cell death and immune response, Kinases

## Abstract

Caspase-8 activity is required to inhibit necroptosis during embryogenesis in mice. In vitro studies have suggested that caspase-8 directly cleaves RIPK1, CYLD and the key necroptotic effector kinase RIPK3 to repress necroptosis. However, recent studies have shown that mice expressing uncleavable RIPK1 die during embryogenesis due to excessive apoptosis, while uncleavable CYLD mice are viable. Therefore, these results raise important questions about the role of RIPK3 cleavage. To evaluate the physiological significance of RIPK3 cleavage, we generated *Ripk3*^D333A/D333A^ mice harbouring a point mutation in the conserved caspase-8 cleavage site. These mice are viable, demonstrating that RIPK3 cleavage is not essential for blocking necroptosis during development. Furthermore, unlike RIPK1 cleavage-resistant cells, *Ripk3*^D333A/D333A^ cells were not significantly more sensitive to necroptotic stimuli. Instead, we found that the cleavage of RIPK3 by caspase-8 restricts NLRP3 inflammasome activation-dependent pyroptosis and IL-1β secretion when Inhibitors of APoptosis (IAP) are limited. These results demonstrate that caspase-8 does not inhibit necroptosis by directly cleaving RIPK3 and further underscore a role for RIPK3 in regulating the NLRP3 inflammasome.

## Introduction

Necroptosis is activated when the activity of caspase-8 is compromised [1, 2]. This lytic form of cell death is characterised by cell swelling and the rupture of cellular membranes. Intracellular contents, released upon membrane permeabilisation, are thought to act as damage-associated molecular patterns (DAMPs) which can trigger immune and inflammatory responses [3]. Consequently, necroptosis is believed to serve as a backup cell death pathway to eliminate infected cells and alert the immune system when pathogens inhibit caspase-8-mediated apoptosis [4].

Necroptosis is triggered by a variety of stimuli, including cytokines of the TNF superfamily (TNFSF) or ligands of pattern recognition receptors (PRR) [4]. The binding of PRR and TNFSF ligands results in the formation of a complex composed of cFLIP, caspase-8, FADD, RIPK1 and RIPK3. This complex is referred to as complex II when formed through TNFR1 activation and as the RIPoptosome when formed downstream of ligands other than TNF [5–7]. Within the complex II/RIPoptosome, caspase-8/cFLIP heterodimers have been reported to cleave RIPK1 and RIPK3 to suppress necroptosis [8, 9]. Additionally, CYLD, which deubiquitinates RIPK1 and favours the formation of the complex II/RIPoptosome, has also been proposed as a caspase-8 substrate to block necroptosis [10–12]. Consequently, in the absence of caspase-8 activity, deubiquitinated and non-cleaved RIPK1 and RIPK3 undergo higher-order oligomerisation *via* RHIM-RHIM interactions leading to their activation and autophosphorylation [13–17]. Subsequently, RIPK3 phosphorylates MLKL, causing its conformational change, oligomerisation, translocation to the plasma membrane, and eventual membrane rupture [18–22].

The rescue of the midgestational lethality to birth of catalytically inactive caspase-8 mice, *Casp8*^C362A/C362A^, by *Mlkl*^-/-^ mice supports the idea that caspase-8 must cleave other proteins to prevent necroptosis activation during embryogenesis [23, 24]. However, recent findings have challenged our knowledge of caspase-8’s necroptotic substrates and their cleavage sites. Mice expressing CYLD D215A develop normally, suggesting that cleavage of CYLD at D215 is not essential to repress necroptosis [25]. cFLIP, another caspase-8 substrate that regulates necroptosis, did not undergo lethal necroptosis when its cleavage was genetically blocked in mice [25, 26]. Furthermore, we and others have shown that rather than just inhibiting necroptosis, caspase-8-mediated RIPK1 cleavage is a mechanism to dissociate complex II/RIPoptosome to block apoptosis and autoinflammation [25, 27–30]. Therefore, RIPK3 might be the primary necroptotic caspase-8 substrate supporting the current model of necroptosis repression. However, RIPK3 cleavage has only been demonstrated in overexpression systems [31]. This prompted us to investigate its physiological role using cleavage resistant RIPK3 mutant mice. Our study revealed that, surprisingly, the cleavage of RIPK3 at D333 by caspase-8 does not inhibit necroptosis during development. Interestingly, we found that RIPK3 restricts NLRP3-dependent pyroptosis and IL-1β secretion by limiting caspase-1 activation when IAPs are inhibited.

## Results

### RIPK3 cleavage does not prevent necroptosis

**Fig. 1:**
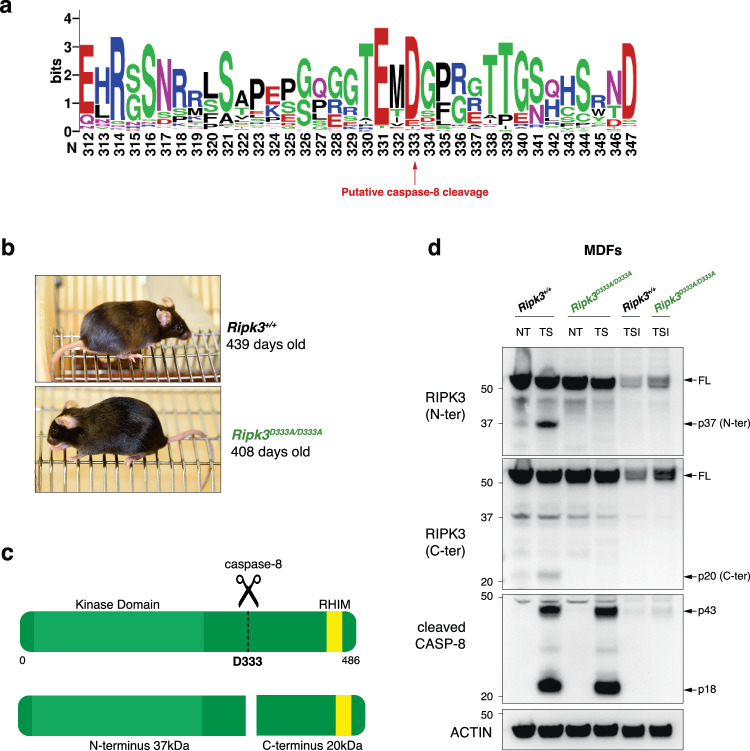
Genetic blockade of caspase-8-mediated RIPK3 cleavage does not lead to spontaneous necroptosis in vivo. **a** WebLogo demonstrating high conservation of the caspase-8-mediated cleavage motif in RIPK3 (mouse numbering) in 94 vertebrate species. **b** Representative pictures of ~400 days old *Ripk3*^*+/+*^ and *Ripk3*^*D333A/D333A*^ mice. **c** Schematic representation of RIPK3 cleavage fragments. **d** Western blot analysis of caspase-8-mediated cleavage of RIPK3 in lysates from MDFs treated with 3 h of TS and TSI. NT, non-treated; TS, 100 ng ml^−1^ TNF (T) plus 1 μM Smac-mimetic compound A (S); TSI, TS plus 10 μM caspase inhibitor IDN-6556 (I). Results are representative of 3 independent experiments performed with 3 independent biological replicates.

*Casp8*^-/-^ and *Casp8*^C362A/C362A^ mice are lethal due to uncontrolled RIPK3-dependent necroptosis [9, 23, 24, 32, 33]. A previous in vitro study showed that RIPK3 is cleaved by caspase-8 at mouse D333 (human D328), located within a highly conserved caspase-8 cleavage motif [31] (Fig. 1a). If caspase cleavage of RIPK3 at D333 is required to prevent necroptosis during mouse embryogenesis, then genetically blocking this cleavage should mimic the lethality observed in *Casp8*^-/-^ and *Casp8*^C362A/C362A^ mice. Yet, *Ripk3*^D333A/D333A^ mice were born at the expected Mendelian frequency, were fertile and appeared normal over a span of 400 days (Fig. 1b). Upon TNF plus Smac-mimetic (CompA [34]; TS) treatment, which cleaved and activated caspase-8, we detected the N- and C-terminus RIPK3 cleavage fragments of the predicted size in wild-type mouse dermal fibroblasts (MDFs; Fig. 1c, d). These fragments were absent in TS-treated *Ripk3*^D333A/D333A^ MDFs and wild-type cells treated with the caspase inhibitor IDUN (TSI) confirming that caspase-8 cleaves RIPK3 at D333 (Fig. 1d). While both RIPK1 and CYLD can be cleaved at other sites when their caspase-8 cleavage sites are mutated [25, 27], we did not find any evidence for an alternative cleavage site in *Ripk3*^D333A/D333A^ MDFs (Fig. 1d). These findings indicate that caspase-8-mediated cleavage of RIPK3 at D333 does not limit lethal necroptosis during development or in a steady state.

**Fig. 2:**
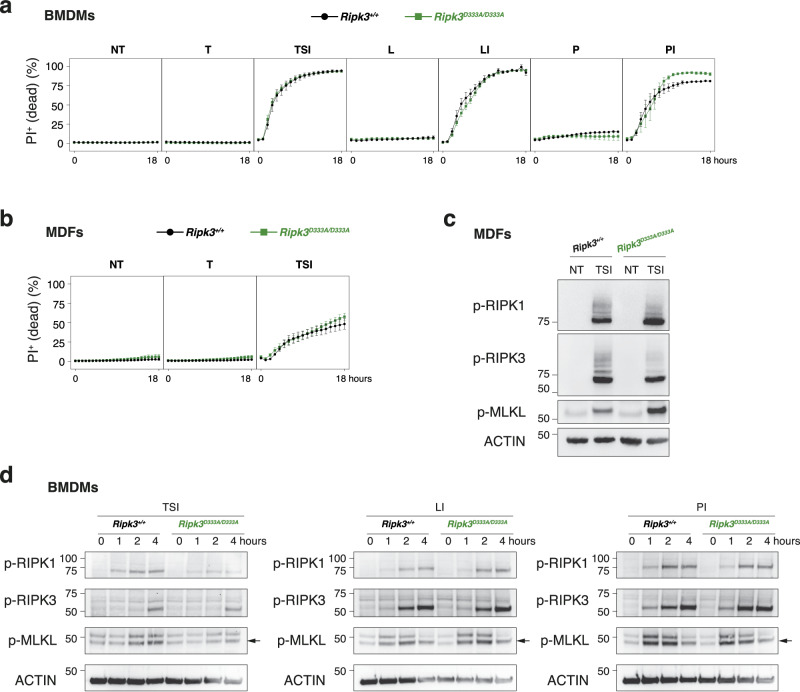
Genetic blockade of caspase-8-mediated RIPK3 cleavage does not increase necroptosis in vitro. Cell death of BMDMs (**a**) and MDFs (**b**) monitored by time-lapse imaging of propidium iodide (PI) staining over 18 h. NT, non-treated; T, 100 ng ml^−1^ TNF; S, 100 nM Smac-mimetic compound A; I, 5 μM caspase inhibitor IDN-6556; L, 25 ng ml^−1^ LPS; P, 5 μg ml^−1^ poly(I:C). Data are represented as mean + SEM of *N* = 3 independent biological replicates per genotype. Graphs are representative of 3 independent experiments performed with 3 independent biological replicates each time. Western blot of MDFs (**c**) treated as in (**b**) for 2 h, and of BMDMs lysates (**d**) treated as in (**a**) for the indicated time. Results are representative of 2 independent experiments performed with 2 independent biological replicates.

We next hypothesised that the cleavage of RIPK3 might nevertheless inhibit necroptosis upon inflammatory challenges. To test this hypothesis, we examined the response of *Ripk3*^D333A/D333A^ cells to TNF and TLR-induced cell death. In contrast to the *Casp8*^-/-^ and non-cleavable RIPK1 cells (*Ripk1*^D325A/D325A^) [9, 25, 27, 30, 35], *Ripk3*^D333A/D333A^ cells did not exhibit sensitivity to TNF alone (Fig. 2a, b). Caspase-8 has been proposed to suppress RIPK3-dependent necroptosis following TLR3/4 engagement [36, 37]. However, we found that the *Ripk3*^D333A/D333A^ bone marrow-derived macrophages (BMDMs) did not undergo cell death after LPS (TLR4 ligand) or poly(I:C) (TLR3 ligand) treatment (Fig. 2a). Despite expectations that non-cleavable RIPK3 would have an enhanced scaffolding function and increase sensitivity to TNF and TLR-induced necroptosis when caspase-8 is inhibited, we did not observe accelerated cell death in *Ripk3*^D333A/D333A^ cells compared to wild-type cells, when treated with IDUN plus low doses of TNF/Smac-mimetic, LPS or poly(I:C) (Fig. 2a, b). Consistently, we did not detect significant changes in the phosphorylation status of RIPK1, RIPK3 and MLKL between wild-type and *Ripk3*^D333A/D333A^ cells in response to necroptotic stimuli, even at early time points (Fig. 2c, d).

### RIPK3 cleavage does not limit the apoptotic scaffolding function of RIPK3

**Fig. 3:**
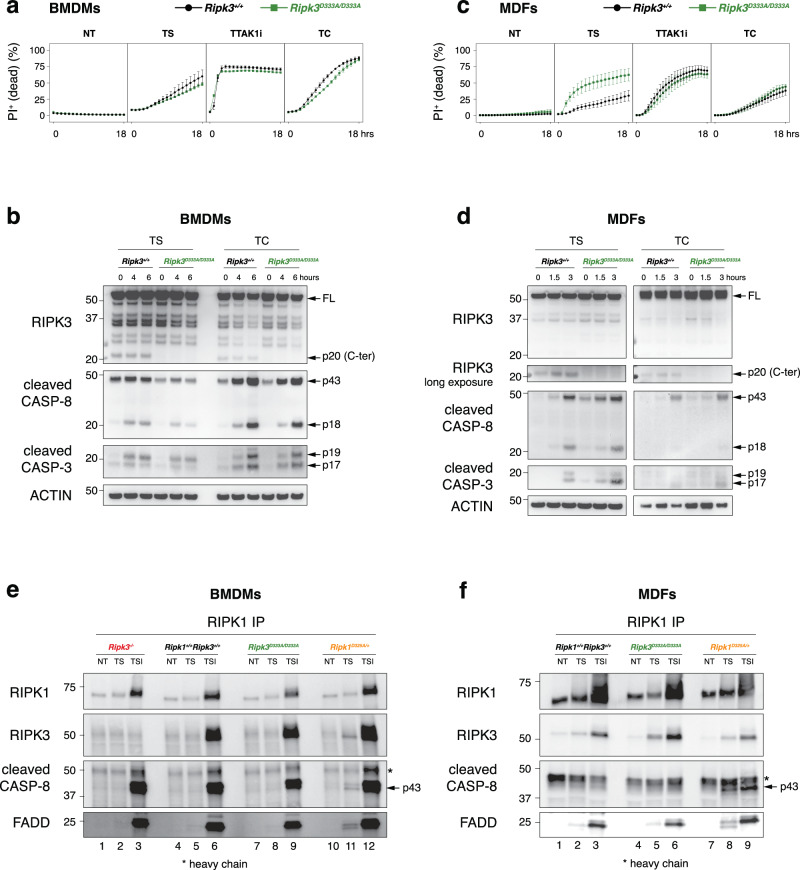
Genetic blockade of caspase-8-mediated RIPK3 cleavage increases apoptosis in vitro. Cell death of BMDMs (**a**) and MDFs (**c**) monitored by time-lapse imaging of propidium iodide (PI) staining over 18 h. NT, non-treated; T, 100 ng ml^−1^ TNF; S, 100 nM Smac-mimetic compound A; TAK1i, 100 nM TAK1 inhibitor; C, 10 μg ml^−1^ cycloheximide. Data are represented as mean + SEM of *N* = 3 independent biological replicates per genotype. Graphs are representative of 3 independent experiments performed with 3 independent biological replicates each time. Western blot of BMDMs (**b**) treated as in (**a**), and of MDFs (**d**) treated as in (**c**). Results are representative of 2 independent experiments performed with 2 independent biological replicates. BMDMs (**e**) and MDFs (**f**) were treated with either 100 ng ml^−1^ TNF and 1 μM Smac-mimetic compound A (TS); or with 100 ng ml^−1^ TNF, 500 nM Smac-mimetic compound A, and 5 μM caspase inhibitor IDN-6556 (TSI). BMDMs were treated with TS for 3 h and with TSI for 1.5 h, while MDFs were treated with TS for 1.5 h and with TSI for 1 h. Immunoprecipitation was performed using anti-RIPK1 antibody. Results are representative of 2 independent experiments performed with 2 independent biological replicates.

RIPK3 has been proposed to act as a scaffold within complex II/Ripoptosome to increase apoptosis [27, 38–41]. We therefore examined whether non-cleavable RIPK3 amplifies its scaffolding function, thereby increasing the susceptibility of *Ripk3*^D333A/D333A^ cells to apoptosis. Cells were treated with TNF in combination with Smac-mimetic (TS), TAK1 inhibitor (TTAK1i) or Cycloheximide (TC), all known to induce complex II formation. In BMDMs, genetic inhibition of RIPK3 cleavage did not alter their sensitivity to TS, TTAK1i or TC-induced apoptosis when compared to wild-type cells (Fig. 3a). Consistent with this, cleavage and activation of caspase-8 and caspase-3 were comparable between wild-type and *Ripk3*^D333A/D333A^ BMDMs upon TS and TC treatments (Fig. 3b). Similarly, *Ripk3*^D333A/D333A^ MDFs were not more sensitive to TTAK1i or TC-induced apoptosis and caspase-8 and -3 processing, indicative of activation, mirrored that of the wild-type cells upon TC (Fig. 3c, d). However, a slight increase in cell death and caspase-8 and -3 activation was observed in *Ripk3*^D333A/D333A^ MDFs treated with TS compared to wild-type MDFs (Fig. 3c, d). This prompted us to assess if non-cleavable RIPK3 enhances complex II formation in response to TS in fibroblastic cells.

Complex II formation is thought to be transiently formed and swiftly inactivated by various molecular checkpoints to prevent TNF toxicity [25, 27, 30, 42–48]. One such checkpoint involved the cleavage of RIPK1 by caspase-8, which participates in the dissociation of complex II [27]. To determine if non-cleavable RIPK3 could also contribute to the stabilisation of complex II, we immunoprecipitated RIPK1 and used non-cleavable RIPK1 cells (*Ripk1*^D325A/+^) as a positive control. As we previously reported in *Ripk1*^D325A/+^ cells [27], complex II was stabilised upon TS treatment as evidenced by the recruitment of caspase-8, FADD and RIPK1 (Fig. 3e lane 11 & f lane 8). In contrast, in both wild-type and *Ripk3*^D333A/D333A^ cells, complex II could not be detected upon TS treatment (Fig. 3e, f). Similarly, the addition of IDUN did not increase the recruitment of caspase-8 or FADD in *Ripk3*^D333A/D333A^ cells compared to wild-type cells (Fig. 3e, f). Altogether, these findings indicate that the blockade of RIPK3 cleavage does not increase its scaffolding function.

### Blocking RIPK1 cleavage does not further sensitise *Ripk3*^D333A/D333A^ mice to cell death

**Fig. 4:**
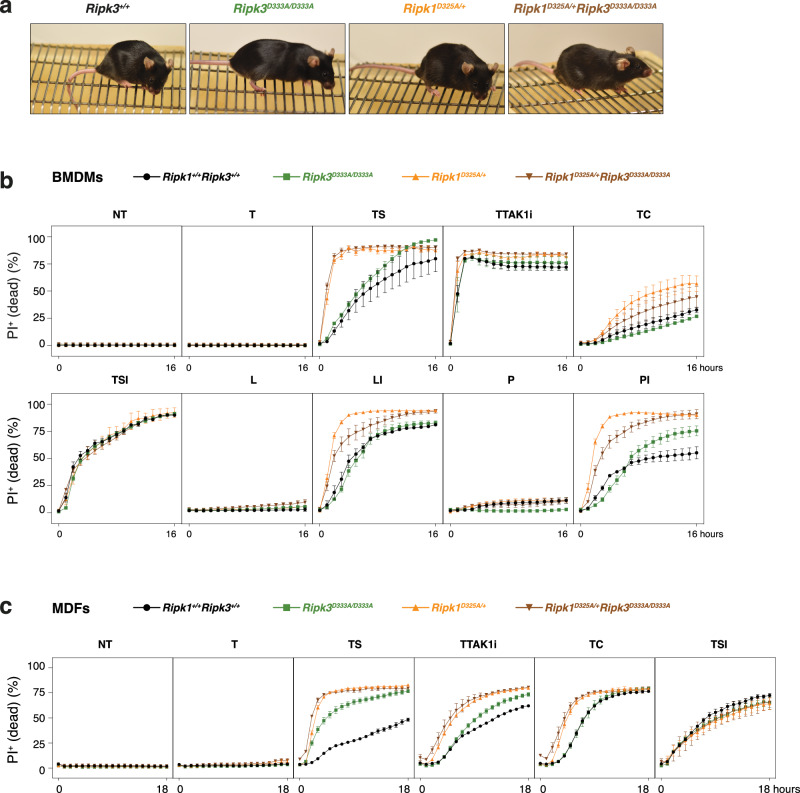
Blocking RIPK1 cleavage does not further sensitise *Ripk3*^D333A/D333A^ mice to cell death. **a** Representative pictures of ~300 days old mice with indicated genotypes. Cell death of BMDMs (**b**) and MDFs (**c**) monitored by time-lapse imaging of propidium iodide (PI) staining over 16 h (**b**) or 18 h (**c**) upon receiving treatments as described in Figs. 2a and  3a. Data are represented as mean + SEM of *N* = 2–3 independent biological replicates per genotype. Graphs are representative of 3 independent experiments performed with 2-3 independent biological replicates each time.

Our results demonstrated that cleavage of RIPK3 at D333 does not prevent necroptosis or limit its scaffolding apoptotic function. However, it could be argued that the cleavage of RIPK1 in *Ripk3*^D333A/D333A^ cells leads to the dissociation of complex II/RIPoptosome, thereby nullifying a role for RIPK3 cleavage. To test this hypothesis, we crossed *Ripk3*^D333A/D333A^ mice to *Ripk1*^D325A/+^ mice and found that *Ripk1*^D325A/+^*Ripk3*^D333A/D333A^ mice were viable, fertile and did not exhibit any overt phenotypic abnormalities for over 300 days of age (Fig. 4a). As previously shown [25, 27, 28, 30], *Ripk1*^D325A/+^ BMDMs and MDFs displayed increased sensitivity to TNFR1-induced apoptosis, except for TTAK1i-treated *Ripk1*^D325A/+^ BMDMs which were as sensitive as the wild-type cells (Fig. 4b, c). *Ripk1*^D325A/+^ BMDMs were also more sensitive to TLR3/4-induced necroptosis (Fig. 4b). However, genetic blockade of RIPK3 cleavage did not further sensitise *Ripk1*^D325A/+^ cells to either apoptosis or necroptosis induced by TNF, LPS or poly(I:C) (Fig. 4b, c).

### RIPK3 cleavage limits NLRP3 activation

**Fig. 5:**
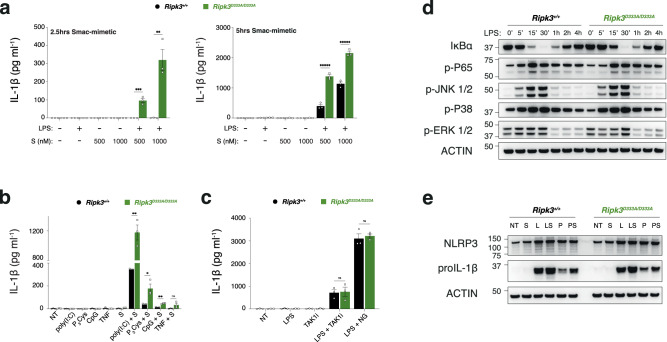
RIPK3 cleavage modulates IL-1β production in vitro. BMDMs were primed for 3 h with 25 ng ml^−1^ LPS (**a**, **c**) or other TLR ligands (**b**) with the following concentrations: 5 μg ml^−1^ poly(I:C); 2 μg ml^−1^ P_3_Cys; 2 μg ml^−1^ CpG; 100 ng ml^−1^ TNF. With the exception of time/dose-dependent compound A treatments in (**a**), BMDMs were treated for 5 h post-priming using 500 nM compound A (**b**) or 100 nM TAK1 inhibitor (**c**). Treatment using nigericin (NG; 5 μM) post-priming in (**c**) was 45 min. Cell supernatants were assayed for IL-1β by ELISA after treatment with indicated compounds. Data are represented as mean + SEM of *N* = 3 independent biological replicates per genotype. Graphs are representative of 3 independent experiments performed with 3 independent biological replicates each time. *P < 0.05, **P < 0.01, ***P < 0.005, *****P < 0.0005, ns not statistically significant. **d** Western blot of BMDMs upon 25 ng ml^−1^ LPS time-course. **e** BMDMs were primed for 3 h either with 25 ng ml^−1^ LPS or 5 μg ml^−1^ poly(I:C), followed by 5 h of 500 nM compound A. Cell lysates were analysed by western blot. **d**, **e** Results are representative of 3 independent experiments performed with 3 independent biological replicates.

The activation of the RIPoptosome in TLR-primed macrophages can trigger robust RIPK3-caspase-8 signalling and activation of the NLRP3 inflammasome, resulting in caspase-1-dependent IL-1β secretion [49–55]. To investigate the role of RIPK3 cleavage in the NLRP3 inflammasome activation, we treated LPS-primed BMDMs with the Smac-mimetic, compound A, a known inducer of RIPK3-dependent NLRP3 activation [50, 51, 53, 55]. Consistent with previous reports, LPS/Smac-mimetic resulted in IL-1β secretion in wild-type BMDMs. Remarkably, in contrast to *Ripk3*^−/−^ BMDMs, *Ripk3*^D333A/D333A^ cells exhibited higher levels of IL-1β release upon LPS/S, in a time and dose-dependent manner (Fig. 5a and Supplementary Fig. 1a). Notably, Smac-mimetic treatment of *Ripk3*^D333A/D333A^ BMDMs primed with other TLR ligands or TNF also led to greater IL-1β secretion compared to wild-type BMDMs (Fig. 5b). In contrast, when LPS-primed BMDMs were treated with the RIPK3-independent NLRP3 activators Nigericin or TAK1 inhibitor, similar levels of IL-1β secretion were observed in both wild-type and *Ripk3*^D333A/D333A^ BMDMs (Fig. 5c).

It has been reported that ectopic expression or forced dimerisation of RIPK3 can activate NF-κB [56–59]. We hypothesised that non-cleavable RIPK3 might accumulate upon TLR treatment and, similar to RIPK3-overexpressing cells, increase NF-κB activation and inflammasome priming. However, treatment with LPS or poly(I:C) did not affect NF-κB activation (Fig. 5d). Furthermore, the levels of NLRP3, caspase-1 and pro-IL-1β were comparable between wild-type and *Ripk3*^D333A/D333A^ after LPS treatment (Fig. 5e). This suggested that the increased IL-1β in non-cleavable RIPK3 cells compared with wild type cells was not due to a priming defect.

**Fig. 6:**
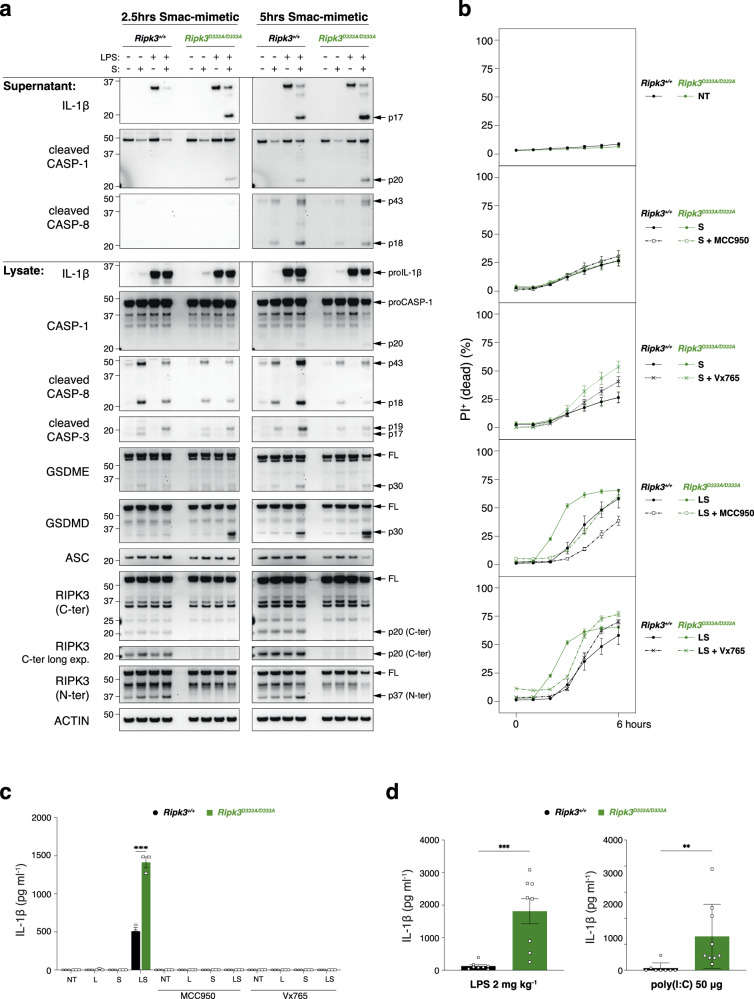
RIPK3 cleavage modulates pyroptosis. **a** BMDMs were primed for 3 h with 25 ng ml^−1^ LPS, then treated for 2.5 h or 5 h with 500 nM Smac-mimetic compound A. Both cell lysates and supernatants were analysed by western blot. Results are representative of 3 independent experiments performed with 3 independent biological replicates. **b** Cell death of BMDMs monitored by time-lapse imaging of propidium iodide (PI) staining over 6 h upon priming for 3 h with 25 ng ml^−1^ LPS, followed by treatment using 500 nM Smac-mimetic compound A+/− MCC950 (5 μM) or Vx765 (10 μM). Data are represented as mean + SEM of *N* = 3 independent biological replicates per genotype. Graphs are representative of 3 independent experiments performed with 3 independent biological replicates each time. **c** BMDMs were primed for 3 h with 25 ng ml^−1^ LPS and then treated for 2.5 h with 500 nM compound A+/− MCC950 (5 μM) or Vx765 (10 μM). Cell supernatants were assayed for IL-1β by ELISA after treatment with indicated compounds. Data are represented as mean + SEM of *N* = 3 independent biological replicates per genotype. Graphs are representative of 3 independent experiments performed with 3 independent biological replicates each time. **d** Mouse littermates were challenged with either LPS (2 mg kg^−1^) or poly(I:C) (50 μg) via intraperitoneal injection. Mouse serum was collected 2 h after treatment and assayed for IL-1β by ELISA. Data are represented as mean + SEM of *N* = 8 mice (LPS) or *N* = 9 mice (poly(I:C)) per genotype performed twice independently. **c**, **d** **P < 0.01, ***P < 0.005.

Next, we examined whether the increase in IL-1β secretion in response to LPS/S correlated with caspase activation. Consistent with the higher levels of IL-1β in *Ripk3*^D333A/D333A^ BMDMs, after 2.5 h of Smac-mimetic treatment, we observed cleavage of caspase-1, GSDMD and IL-1β in *Ripk3*^D333A/D333A^ BMDMs and not in wild-type cells (Fig. 6a). In contrast, the level of caspase-8, -3 and GSDME cleavage were slightly reduced in *Ripk3*^D333A/D333A^ BMDMs compared to wild-type cells after LPS/Smac-mimetic treatment (Fig. 6a). After 5 h of Smac-mimetic treatment, levels of caspase-1, GSDMD processing in *Ripk3*^D333A/D333A^ BMDMs were comparable to those observed in wild-type BMDMs (Fig. 6a). The reduction of caspase-8, -3 and GSDME cleavage were more pronounced in the *Ripk3*^D333A/D333A^ BMDMs after 5 h of Smac-mimetic compared to wild-type BMDMs (Fig. 6a). While loss of RIPK3 blocked LPS/Smac-mimetic (LS)-induced cell death, the increase in caspase-1 and GSDMD cleavage in the *Ripk3*^D333A/D333A^ BMDMs was accompanied by an accelerated cell death after 3 h of LS treatment (Fig. 6b and Supplementary Fig. 1b). Importantly, both the NLRP3 inhibitor MCC950 [60] and the caspase-1 inhibitor Vx765 significantly reduced cell death and IL-1β secretion in the *Ripk3*^D333A/D333A^ BMDMs (Fig. 6b, c).

Our in vitro results demonstrate that genetic blockade of RIPK3 cleavage dominantly exaggerates TLR-induced NLRP3 inflammasome-dependent IL-1β secretion. We therefore challenged *Ripk3*^D333A/D333A^ mice intraperitoneally with sublethal doses of LPS or poly (I:C) and measured serum IL-1β levels after 2 h. Consistent with our in vitro findings, *Ripk3*^D333A/D333A^ mice had markedly enhanced serum IL-1β levels when compared to wild-type mice (Fig. 6d), confirming that cleavage of RIPK3 is critical to limit TLR-induced NLRP3 inflammasome activity. Collectively, our data demonstrate that cleavage of RIPK3 is not essential to limit necroptosis but rather represses TLR-induced NLPR3-dependent IL-1β production when IAPs are in low abundance or inhibited.

## Discussion

The proximity of caspase-8, RIPK1 and RIPK3 within complex II/RIPoptosome, as well as the rescue of *Casp8*-deficient and catalytically inactive mice by loss of *Ripk3* or *Mlkl* supports the notion that caspase-8 cleaves RIPK3 to prevent necroptosis. However, unlike the non-cleavable RIPK1 mice, our findings reveal that the genetic blockade of RIPK3 cleavage at D333 does not lead to uncontrolled lethal necroptosis. These results suggest the existence of other molecular mechanisms that may substitute or cooperate with RIPK3 cleavage in suppressing necroptosis. One proposed mechanism could be the regulation of RIPK3’s necroptotic function through ubiquitylation [61–67]. Therefore, it is plausible that an E3 ligase or a deubiquitinase cleaved by caspase-8 inhibits RIPK3-dependent necroptosis. While CYLD could be a potential candidate a recent study showed that its cleavage is not required to inhibit necroptosis during embryogenesis [25]. If there is redundancy, the generation of *Ripk3*^D333A/D333A^*Cyld*^D215A/D215A^ double mutant mice will allow this hypothesis to be tested.

Akin to necroptosis, genetically blocking RIPK3 cleavage had little to no impact on its apoptotic scaffolding function. This is somewhat surprising given that the increased activation of caspase-8 observed in uncleavable RIPK1 cells was markedly reduced on a *Ripk3*^*-/-*^ background [25, 27]. These findings raise the question of why the RIPK3 cleavage site is conserved during evolution, even in organisms that do not have the capability to undergo necroptosis. Our finding that RIPK3 cleavage plays a role in limiting IL-1β secretion offers a potential explanation. These results reinforce the previously described involvement of RIPK3 in driving NLPR3 inflammasome activation [49–55]. Significantly, our study provides new insights by revealing that RIPK3 cleavage is a negative feedback mechanism to restrain excessive NLPR3 inflammasome activation in situations when IAPs are scarce. These findings hold relevance for individuals with XIAP deficiency, wherein degradation of cIAPs through the TLR-TNFR2 pathway could trigger RIPK3-dependent inflammasome activation [55, 68]. Furthermore, our results shed light on the role of caspase-8 and RIPK3 in development and why the loss of *Ripk3* extends the lifespan of catalytically inactive caspase-8 (*Casp8*^C362A/C362A^) mice when compared to the loss of *Mlkl* [23, 24]. We can conclude that caspase-8 exerts repression over necroptosis during embryogenesis through multiple mechanisms and restrain pyroptosis after birth *via* cleavage of RIPK3.

In non-cleavable RIPK3 BMDMs, we did not observe an increase in caspase-8 processing in response to LPS/Smac-mimetic, suggesting that non-cleavable RIPK3 did not enhance RIPoptosome formation. This aligns with our results when cells were treated with TS or TSI treatment. However, it’s worth noting RIPK3 can drive mitochondrial respiration and ROS production [15], and ROS have previously been linked to RIPK3-driven NLRP3 responses [51]. This may explain the increased TLR-RIPK3-NLRP3 signalling observed in RIPK3 D333A mutant macrophages, although this idea requires further investigation.

## Materials and methods

### Mice

All mouse studies complied with relevant ethical regulations and were approved by the Walter and Eliza Hall Institute and the Peter MacCallum Cancer Centre Animal Ethics Committees. The *Ripk1*^*D325A/+*^ and *Ripk3*^*D333A/D333A*^ mice were generated by the MAGEC laboratory (WEHI, Australia) on a C57BL/6 J background. To generate *Ripk1*^*D325A/+*^ mice, 20 ng μl^−1^ of *Cas9* mRNA, 10 ng μl^−1^ of sgRNA (ATTTGACCTGCTCGGAGGTA) and 40 ng μl^−1^ of the oligo donor (tgtcttctcattacagAAAGAGTATCCAGATCAAAGCCCAGTGCTGCAGAGAATGTTTTCACTGCAGCATGCCTGTGTACCATTACCTCCGAGCAGGTCAAATTCAGgtaactcacctattcgttcatttgcatactcgctca) (in which uppercase bases denote exons; lowercase bases denote intron sequences) were injected into the cytoplasm of fertilised one-cell stage embryos generated from wild-type C57BL/6 J breeders. To generate *Ripk3*^*D333A/D333A*^ mice, 20 ng μl^−1^ of *Cas9* mRNA, 10 ng μl^−1^ of sgRNA (GCACAGAAATGGATTGCCCG) and 40 ng μl^−1^ of oligo donor (CACAGAAGCAGCGGCAGAAACTTGTCTGCCAGAGAGCCAAGCCAAAGAGGCACAGAAATGGcgTGCCCGAGGGAAACCATGGTTTCTAAAATGCTGGACCGCCTGCATTTGGAGGAACCCTCCGGACCAGTT) were injected into the cytoplasm of fertilised one-cell stage embryos generated from wild-type C57BL/6J breeders. Twenty-four hours later, two-cell stage embryos were transferred into the uteri of pseudo-pregnant female mice. The *Ripk1*^*D325A/+*^*Ripk3*^*D333A/D333A*^ double mutant mice were generated by crossing *Ripk3*^*D333A/D333A*^ mice to *Ripk1*^*D325A/+*^ mice. Viable offspring were genotyped by next-generation sequencing.

### Reagent

The Smac-mimetic compound A and the caspase inhibitor IDN-6556 (Idun Pharmaceuticals) were synthesised by TetraLogic Pharmaceuticals. The RIPK3 inhibitor GSK’872 was from Calbiochem. The TAK1 inhibitor (5Z)-7-oxozeaenol was from Tocris Bioscience. Cycloheximide and nigericin were from Sigma. The NLRP3 inhibitor MCC950 was purchased from Merck. Caspase-1-specific inhibitor Vx765 was from MedChemExpress. Ultrapure LPS-EB, poly(I:C), P_3_Cys, and CpG were purchased from Invivogen.

### Cells

MDFs were isolated from mouse tails, followed by SV40 transformation and subsequently cultured in complete Dulbecco’s Modified Eagle Medium (DMEM) containing 8% foetal bovine serum (FBS), 100 U ml^−1^ penicillin, and 100 µg ml^−1^ streptomycin at 37 °C, 10% CO_2_. To generate BMDMs, bone marrow cells were flushed from murine femoral and tibial bones and cultured in 15 cm Petri dishes for 6 days in complete DMEM (containing 8% FBS, 100 U ml^−1^ penicillin, and 100 µg ml^−1^ streptomycin) supplemented with 20% L929 conditioned media (containing Macrophage Colony-stimulating Factor, M-CSF) at 37 °C and 10% CO_2_. MDFs, BMDMs, and 293 T cells (ATCC) used to produce SV40 viruses were also tested for mycoplasma but not authenticated.

### Cell death

MDFs were plated at 1 × 10^4^ cells per well (96-well flat-bottom plate, Greiner) in complete DMEM on the same day as treatment. BMDMs were plated at 1 × 10^5^ cells per well (96-well) in complete DMEM supplemented with 20% L929 conditioned media, either the day before or on the same day as the treatment. Media were replaced and cells were labelled with SPY505-DNA (Spirochrome SC-101, 1:1000 dilution) for 1 h at 37 °C, 10% CO_2_ before imaging and appropriate treatment compounds were added 5 min prior to the initiation of imaging. Percentage cell death was assayed every 30 min or 1 h by time-lapse imaging using the IncuCyte live cell analysis imaging (Sartorius) for a total of 16–18 h under 5% CO_2_ and 37 °C conditions. Dead cells were identified by propidium iodide (PI, 0.25 μg ml^−1^) staining and the percentage of cell death was calculated as the ratio of PI-positive cells to total cells stained with SPY505-DNA (Spirochrome SC-101).

### Immunoblotting

Cell lysates and supernatants (denatured and reduced with SDS loading buffer) were separated on 4–12% gradient SDS–polyacrylamide gels (Invitrogen and Bio-Rad) and transferred to polyvinylidene fluoride (PVDF) membranes (Millipore) for detection. Membranes were blocked with 5% w/v bovine serum albumin (BSA, Sigma) in Tris-buffered saline containing 0.1% Tween-20 (TBS-T) for 1 h at room temperature and were probed overnight with primary antibodies at 4 °C. All primary antibodies were, unless otherwise stated, diluted 1/1000 in TBS-T containing 5% w/v BSA and 0.05% sodium azide. Relevant secondary antibodies conjugated with horseradish peroxidase were diluted in TBS-T containing 5% w/v skim milk and were applied for 1 h at room temperature. All membranes were washed three times (10 min each) in-between antibody incubations, then developed using ECL (Millipore) and imaged using the iBright Imaging System (ThermoScientific). Primary antibodies used include β-actin (1/10000 dilution, Sigma; A-1978), RIPK1 (Cell Signaling; 3493), RIPK3 C-terminal (Axxora; PSC-2283-c100), RIPK3 N-terminal (WEHI; 1H12), phospho-RIPK1 Ser166 (Cell Signaling; 31122), phospho-RIPK3 (1/2500 dilution, a gift from Genentech), cleaved caspase-8 Asp387 (Cell Signaling; 8592), caspase-1 (Adipogen; AG-20B-0042), cleaved caspase-3 Asp175 (Cell Signaling; 9661), GSDMD (Abcam; ab209845), GSDME (Abcam; ab215191), NLRP3 (Adipogen; AG-20B-0014), ASC (Adipogen; AG-25B-0006), IL-1β (R&D Systems; AF-401-NA), phospho-MLKL Ser345 (Abcam; ab196436), FADD (Enzo; ADI-AAM-212), IκBα (Cell Signaling; 9242), phospho-p65 Ser536 (Cell Signaling; 3033), phospho-JNK1/2 Thr183/Tyr185 (Cell Signaling; 4668), phospho-p38 Thr180/Tyr182 (Cell Signaling; 4511), and phospho-ERK1/2 Thr202/Tyr204 (Cell Signaling; 9101). Uncropped western blots are provided in Supplementary Fig. 2.

### Immunoprecipitation

Ten million (1 × 10^7^) cells were seeded in 15 cm tissue culture dishes and treated accordingly. After the indicated treatments, cells were lysed in DISC lysis buffer (150 mM sodium chloride, 2 mM EDTA, 1% Triton X-100, 10% glycerol, 20 mM Tris, pH 7.5, Roche complete protease inhibitor cocktail, Roche phosSTOP phosphatase inhibitor). Proteins were immunoprecipitated with 20 μl of protein A Sepharose plus 8 μg of RIPK1 antibody (Cell Signaling; 3493) with overnight rotation at 4 °C. Beads were washed four times in DISC and samples eluted by boiling in 60 μl 1× SDS loading dye.

### ELISA

Mouse serum and cellular supernatants from BMDMs were analysed by ELISA for IL-1β content (R&D Systems) according to the manufacturer’s instructions.

### In vivo TLR challenge

Eight-to-twelve-week-old mouse littermates received intraperitoneal injection of either 2 mg kg^−1^ LPS or 50 μg poly(I:C). Calculations to determine group sizes were not performed, mice were not randomised but were grouped according to genotype. Animal technicians and researchers who performed intraperitoneal injection and ELISA were blinded to the mice genotype and treatment conditions.

### Statistical analyses

An unpaired parametric two-sided *t*-test was used for in vitro data and unpaired nonparametric two-sided Mann-Whitney test was used for in vivo data. *P* values *P < 0.05; **P < 0.01; ***P < 0.005; ****P < 0.001; *****P < 0.0005; ******P < 0.0001 were considered statistically significant.

### Reporting summary

Further information on research design is available in the Nature Research Reporting Summary linked to this article.

### Supplementary information

Supplemental Material

Reporting summary

## Data Availability

The datasets generated during and/or analysed during the current study are available from the corresponding author on reasonable request. Mice generated by this study are available under a material transfer agreement with the Peter MacCallum Cancer Centre.
