# Supplementary material for: RIPK3 cleavage is dispensable for necroptosis inhibition but restricts NLRP3 inflammasome activation
Source: Cell Death Differ. 2024 Mar 21;31(5):662–71. doi: 10.1038/s41418-024-01281-x (PMC11094093; doi:10.1038/s41418-024-01281-x)
Supplement: Supplementary file 1 — Supplemental Material [file 41418_2024_1281_MOESM1_ESM.pdf]

Supplementary material

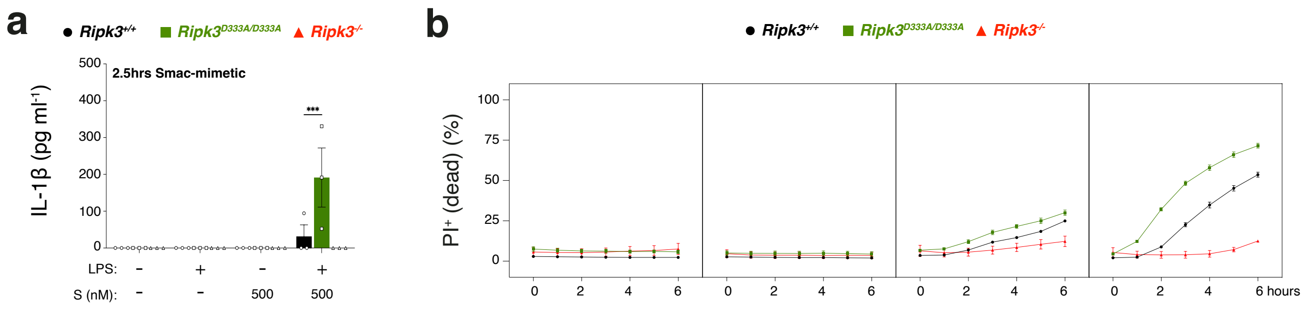

Sup Fig 1 Loss of RIPK3 blocks LPS/SM-induced pyroptosis and IL-1β release. a BMDMs were primed for 3 hours with 25 ng ml^{-1} LPS, then treated for 2.5 hours with 500 nM Smac-mimetic compound A (S). Cell supernatants were assayed for IL-1 β by ELISA after treatment. **b** Cell death of BMDMs monitored by time-lapse imaging of propidium iodide (PI) uptake. Cells were primed for 3 hours with 25 ng ml^{-1} LPS, then treated for 6 hours with 500 nM Smac-mimetic compound A. NT, non-treated; L, LPS; S, Smac-mimetic compound A.

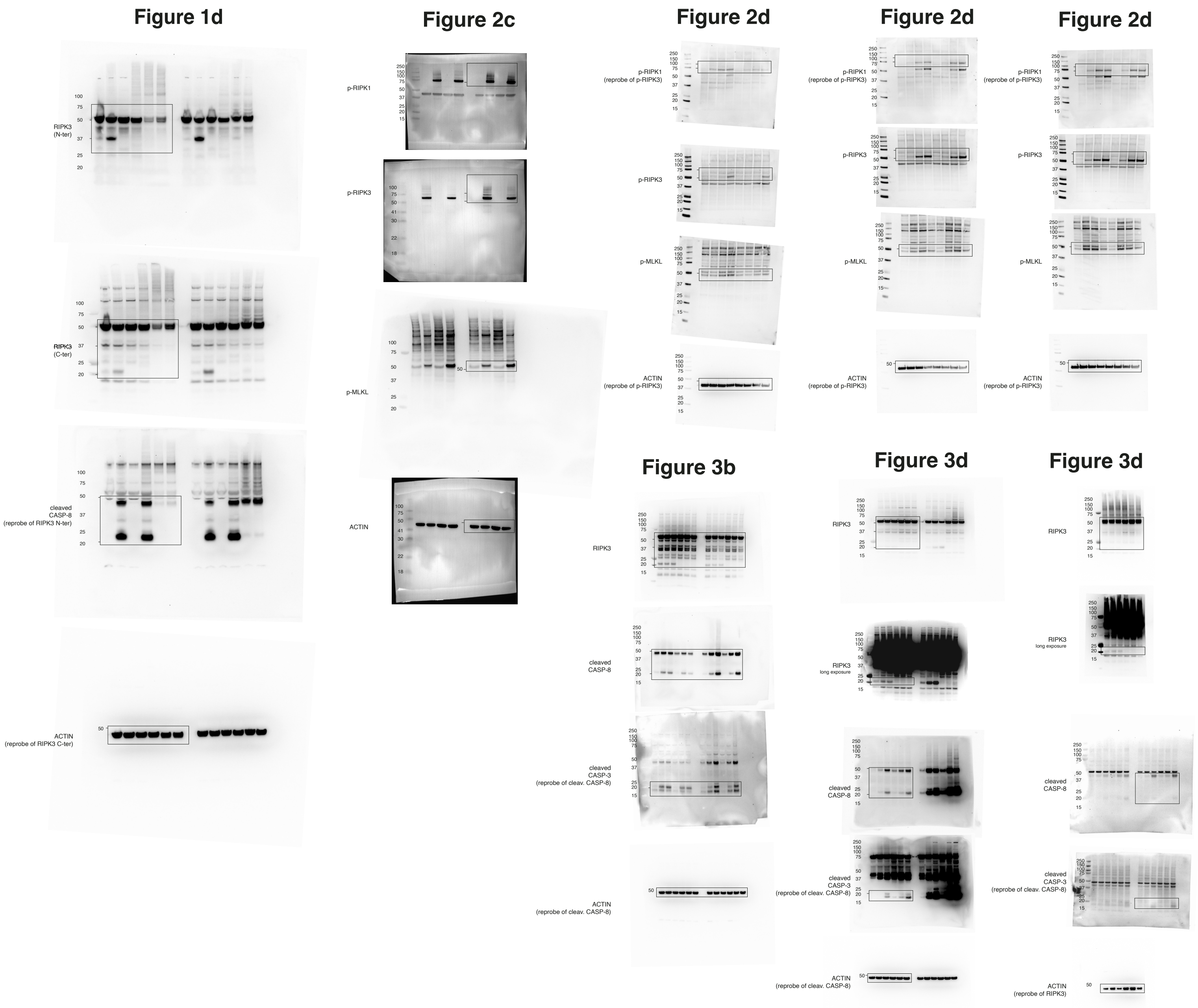

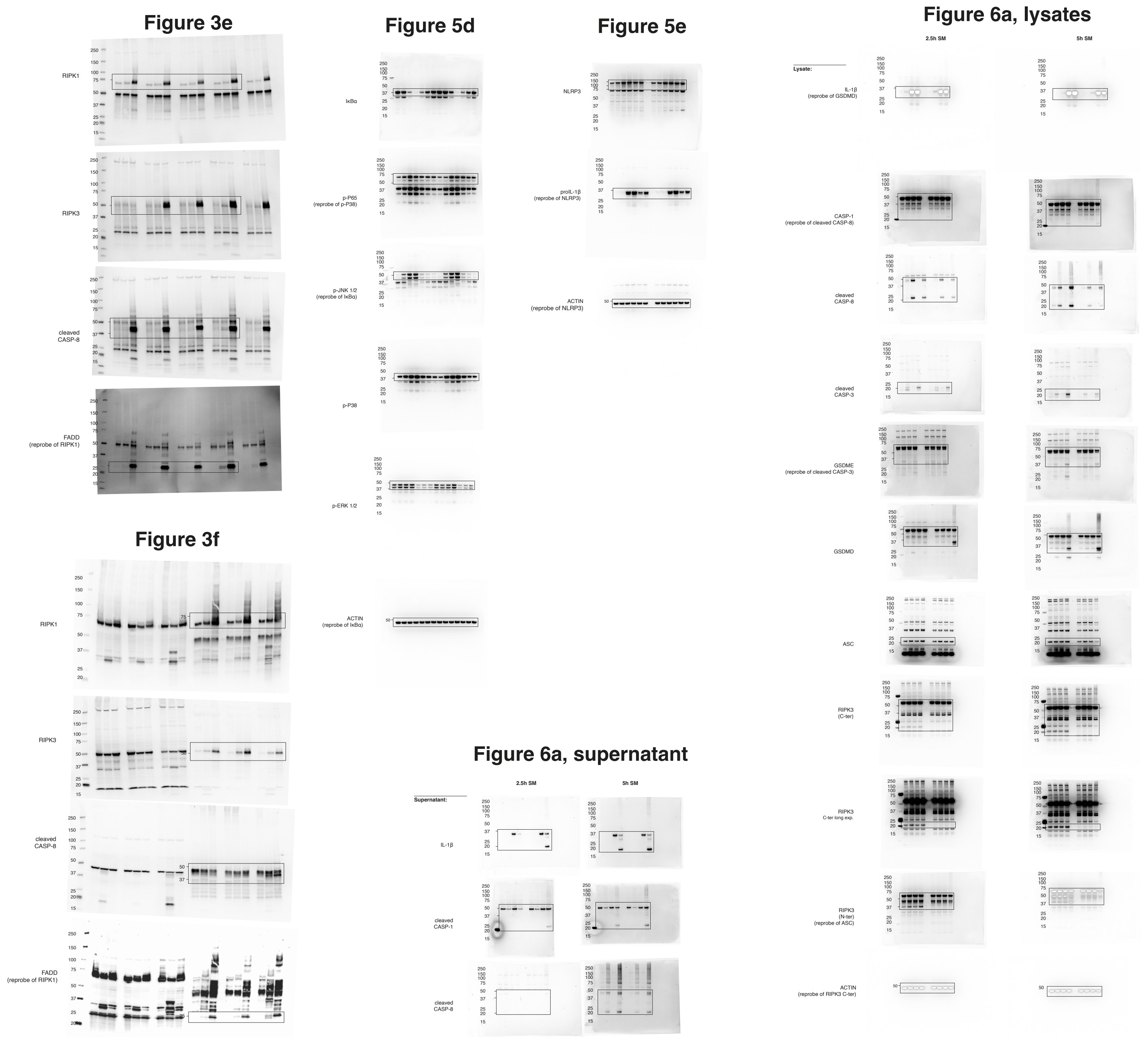

Sup Fig 2 Original data files. Uncropped western blot membranes for each figure.
